# Effect of a multicomponent exercise program and cognitive stimulation (VIVIFRAIL-COGN) on falls in frail community older persons with high risk of falls: study protocol for a randomized multicenter control trial

**DOI:** 10.1186/s12877-022-03214-0

**Published:** 2022-07-23

**Authors:** Juan Luis Sánchez-Sánchez, Cristina Udina, Almudena Medina-Rincón, Mariano Esbrí-Victor, Irene Bartolomé-Martín, Débora Moral-Cuesta, Itxaso Marín-Epelde, Fernanda Ramon-Espinoza, Marina Sánchez- Latorre, Fernando Idoate, Adriana Goñi-Sarriés, Blanca Martínez-Martínez, Raquel Escudero Bonet, Julián Librero, Álvaro Casas-Herrero

**Affiliations:** 1 MOVE-IT Research Group, Department of Physical Education, Faculty of Education Sciences, University of Cadiz, Cadiz, Spain; 2 Health Sciences Department, Universidad Pública de Navarra (UPNA), Pamplona, Spain; 3 Insitut de Viellissement, CHU Toulouse, Gerontopole de Toulouse, Toulouse, France; 4 Parc Sanitari Pere Virgili, Barcelona, Spain; 5 RE-FiT Bcn Research Group, Vall Hebron Research Institute, Barcelona, Spain; 6 Geriatrics Department, Complejo Hospitalario Universitario de Albacete (CHUA), Albacete, Spain; 7 Geriatrics Department, Hospital Universitario de Guadalajara (HUG), Guadalajara, Spain; 8 Geriatrics Department, Hospital Universitario de Navarra (HUN), C/Irunlarrea s/n 31008, Pamplona, Spain; 9 Functional Recovery Unit, Hospital San Juan de Dios (HSJD), Pamplona, Spain; 10 Radiology Department, Mutua Navarra, Pamplona, Spain; 11 Mental Health Network, Navarra Healthcare System, Pamplona, Spain; 12 Navarra Institute for Health Research (IdiSNA), Pamplona, Spain; 13 CIBER of Frailty and Healthy Aging (CIBERFES), Instituto de Salud Carlos III, Madrid, Spain

**Keywords:** Falls, Physical exercise, Cognitive function, Fractures, Frailty

## Abstract

**Background:**

Falls represent important drivers of intrinsic capacity losses, functional limitations and reduced quality of life in the growing older adult’s population, especially among those presenting with frailty. Despite exercise- and cognitive training-based interventions have shown effectiveness for reducing fall rates, evidence around their putative cumulative effects on falls and fall-related complications (such as fractures, reduced quality of life and functional limitations) in frail individuals remains scarce. The main aim of this study is to explore the effectiveness program combining an individualized exercise program and an executive function-based cognitive training (VIVIFRAIL-COGN) compared to usual care in the prevention of falls and fall-related outcomes over a 1-year follow-up.

**Methods:**

This study is designed as a four-center randomized clinical trial with a 12-week intervention period and an additional 1-year follow-up. Three hundred twenty frail or pre-frail (≥ 1 criteria of the Frailty Phenotype) older adults (≥ 75 years) with high risk of falling (defined by fall history and gait performance) will be recruited in the Falls Units of the participating centers. They will be randomized in a 1:1 ratio to the intervention group (IG) or the control group (CG). The IG will participate in a home-based intervention combining the individualized Vivifrail multicomponent (aerobic, resistance, gait and balance and flexibility) exercise program and a personalized executive function-based cognitive training (VIVIFRAIL-COGN). The CG group will receive usual care delivered in the Falls Units, including the Otago Exercise Program. Primary outcome will be the incidence of falls (event rate/year) and will be ascertained by self-report during three visits (at baseline, and 6 and 12 weeks) and telephone-based contacts at 6, 9 and 12 months after randomization. Secondarily, effects on measures of physical and cognitive function, quality of life, nutritional, muscle quality and psychological status will be evaluated.

**Discussion:**

This trial will provide new evidence about the effectiveness of an individualized multidomain intervention by studying the effect of additive effects of cognitive training and physical exercise to prevent falls in older frail persons with high risk of falling. Compared to usual care, the combined intervention is expected to show additive effects in the reduction of the incidence of falls and associated adverse outcomes.

**Trial registration:**

NCT04911179 02/06/2021.

**Supplementary Information:**

The online version contains supplementary material available at 10.1186/s12877-022-03214-0.

## Introduction

### Background

Approximately, 1 out of 3 adults aged 65 or over experience at least one fall per year and among them, 10% suffer a significant injury such as a fracture or traumatic brain injury [1]. These figures rocket in those aged 80 years or more, with half of them falling over the course of a year [2]. Around 10% of falls result in a fracture [3, 4]. In the European Union, it has been estimated that 36,000 older adults die because of a fall yearly, whereas the figures of hospital admission and emergency department visits approximate to 1.5 million and 2.3 million, respectively [5].

Although most fall-related injuries (bruising, lacerations, sprains…) are not severe, they represent important drivers of decline of the intrinsic capacity (physical and cognitive capacities of the individual) [6], functional limitations development, reduced quality of life and the development of psychosocial issues (anxiety, fear of falling and depression) [7, 8]. Therefore, falls constitute a significant sentinel event for older adults by threatening their health and well-being. With the population of older adults increasing worldwide [9], falls will continue to represent an increasing health and economic burden on the public health system. Consequently, prevention of falls has become an international public health priority [10, 11].

Most of the clinical practice guidelines recommend managing older frail adults with repeated falls in specialized Falls and Fracture Units [12]. Ideally management of falls should consist of a multidimensional comprehensive assessment [13] followed by tailored multidomain interventions lead by an interdisciplinary team (physician, nurse, physiotherapist and occupational therapist) and focused to identify and tackle risk factors [12, 14].

Among them, progressive cognitive and physical function decline stand out, both as targets of falls preventive and mitigating interventions, given their tight relation with their occurrence [15–17]. Impairments in the physical domain, such as muscle strength [18], gait performance [19] and balance [20], are strong risk factors for falls, whereas decreases in the cognitive abilities, especially in the executive function domain [21], have been associated with the odds of falling [22] and suffering fall-related injuries [23]. Executive function is a cognitive domain composed of a broad set of cognitive processes integrating information from multitude cortical systems to generate goal-directed behavior [24]. It constitutes an integral function and one of the most important components of daily-living navigating [25]. In addition, robust evidence supports the relationship between cognition and motor function, with common anatomical substrates and neural pathways. Allocated in the prefrontal cortex, executive functions play a key role in neural control of gait [26, 27], especially under challenging circumstances. For instance, when an attention-demanding cognitive task is performed during a walking task, known as dual-task, the performance of either one or both tasks may decrease compared to each individual task as a result of competence for common neural pathways [28]. This motor-cognitive crosstalk also explains the higher risk of falls among persons with cognitive and physical impairment, therefore interventions targeting both might be key to reduce falls risk [29].

Loss of capacities constitute manifestations of the progressive loss of reserve at multi-systemic physiological levels, leading to increased vulnerability when facing stressors, a state that was decades ago termed frailty [30–32]. Frail individuals present with the highest risk of suffering falls and related outcomes [33, 34]. Consequently, frail individuals might benefit the most from interventions oriented to falls prevention trough combined interventions, but the number of studies in this population is limited [35].

In this sense, physical exercise stands out as an effective cornerstone in falls prevention among geriatric patients [13, 36]. Exercise has been shown to concurrently positively impact both physical and cognitive function of older adults [37–40], which might result in the observed reductions in the risk and rate of falls both in community-dwelling [41, 42] and residential populations [43]. In addition, exercise has shown to beneficially impact fall-related issues such as fear of falling [44]. Previous evidence has shown that multicomponent physical exercise programs combining strength and balance exercises might confer the greatest benefits in older adults with high risk of falling [36, 42]. Nevertheless, so far, the features of an exercise program as a single intervention that might maximize effectiveness remain to be elucidated [36].

Given that cognitive impairments are associated with falls occurrence [45], cognitive training, usually involves a guided practice on a set of specific tasks designed to engage targeted cognitive functions [46], might be beneficial in falls prevention [47]. Cognitive training interventions like dual task training, improve attention, executive function and memory in older persons with cognitive decline but also in healthy older adults [48]. A reduced number of emerging studies have shown cumulative effects in fall risk parameters of combined exercise and cognitive training interventions compared to interventions focused exclusively in motor function [49]. Both animal- and human-based studies have shown greater neurophysiological (neurogenesis, neuroplasticity and increased brain blood flow) and functional (physical and cognitive performance) [50, 51] after the combined intervention, compared to single intervention-based approaches [52, 53]. Putatively, programs combining physical exercise and cognitive training focused in executive function tasks might constitute the optimal approach to maximize reductions in fall risk and associated complications [12, 54], but whether available evidence translate to reductions in falls incidence remains to be elucidated [55, 56].

The OTAGO exercise program (OEP), that was developed in the 1960s in New Zealand [57], is considered the gold-standard exercise-based intervention in falls prevention. It consists of several strength, endurance, flexibility and balance exercises supervised by a physical therapist. It has shown to be effective in improving fall-related risk factors [58–60] and preventing falls [61, 62] and related consequences [63, 64]. Notably, several adaptations have been performed since it was firstly designed, leading to a high degree of heterogeneity, with mixed results obtained from different delivery fashions [65–67]. In addition, falls and fall-related outcomes have been poorly registered in available studies, and how exercise programs impact fall-related fractures, healthcare utilization, psychological and social issues remains scarcely investigated [68]. Consequently, well-powered studies with long follow-up periods and comprehensive evaluation of falls consequences are needed in order to capture the real potential of exercise programs in different populations of older adults [42].

Recently, the VIVIFRAIL Project (www.vivifrail.com) was developed by world experts in the field of physical exercise and frailty aiming to provide necessary knowledge to prescribe individualized physical exercise in the prevention of frailty and falls in older persons. Within the VIVIFRAIL philosophy, exercise prescription is based on the functional status and fall risk of the individual, evaluated through performance-based measures [69]. This approach aligns with the recently proposed function-based healthy ageing paradigm [70]. So far, this exercise program has been proven safe and effective to reverse the hospitalization-associated functional decline in very old patients [71] and to promote functional gains in cognitively impaired frail community-dwellers [72], but its effectiveness to reduce falls and health related-consequences, and whether its combination with cognitive training have added effects remain uncertain.

In this context, the main objective of this study is to evaluate the effectiveness of a combined individualized multicomponent exercise and cognitive training (VIVIFRAIL-COGN) program on the reduction in fall-incidence in a sample of community-dwelling frail older adults with high fall risk, compared to usual care (including the OEP). Secondarily, we will investigate the impact on fall-related consequences, such as need for medical assistance, psychological issues (fear of falling and depression), physical and cognitive capacities and quality of life.

## Methods

### Study design and setting

The present study is a multicenter randomized clinical trial to be conducted in the Falls Unit of the geriatrics department of four tertiary hospitals in Spain. Its main aim will be to explore the effectiveness of a multicomponent exercise program combined with a cognitive stimulation program in the reduction of falls and related outcomes among frail older adults. Falls units are specialized clinical environments, to which older adults with recurrent falls are referred from Primary Care or other medical specialties. In these Units, falls etiology and potential management are studied by a multi-disciplinary team. Interventions include physical exercise programs, polypharmacy reduction, environmental adequation in accordance with published international and national guidelines [73].

Patients who meet the inclusion criteria for this study will be randomly assigned to usual care or an intervention consisting in the combination of individualized exercise and cognitive stimulation program. Prior to randomization, the attending geriatrician will review the absolute and relative contraindications to participate in exercise programs and will provide general information about the study. Patient recruitment will begin with the normal visit of the patient to the clinic, in which inclusion criteria will be ascertained and informed consent obtained. Later, subjects will be randomly assigned (as explained later) to either the intervention or the usual care (control group).

### Study participants and eligibility criteria

The study will include outpatients of the Falls Units of the University Hospital of Navarra in Pamplone, the Hospital of Guadalajara, the Hospital Perpetuo Socorro in Albacete and the Parc Sanitari Pere Virgili in Barcelone (all in Spain) recruited between June 2021 and August 2023. Inclusion criteria include:

- Age ≥ 75 years or older
- Referral to the Falls Unit
- Ability to ambulate independently with or without technical aids
- Barthel Index ≥ 60
- Pre-frailty (1–2 criteria) or Frailty according to the Frailty Phenotype by Fried et al. [30]
- High risk of falling defined by one or more of the following criteria

- Gait disorders captured through physical performance measures (Time Up and Go Test ≥ 20 s and/or Usual Gait Speed (GS) < 0,8 m/s [74] 
- ≥ 2 self-reported falls in the previous year
- ≥ 1 self-reported falls requiring medical assistance in the previous year

- Relative/caregiver willingness to supervise the exercise/cognitive stimulation sessions
- Capability and willingness to provide informed consent

### Subjects will be excluded from participation based on the following exclusion criteria

- Unwillingness to either complete the study requirements or to be randomized into the control or the intervention group
- Life expectancy ≤ 3 months
- Terminal illness
- No possibility of follow-up
- Institutionalization or awaiting institutionalization
- Major cognitive disorder according to GDS Reisberg (GDS) classification: 5 or higher
- Severe visual o hearing deprivation
- Any contraindications for physical exercise or testing procedures, including but not limited to:

- myocardial infarction in the past 3 months
- unstable angina pectoris
- uncontrolled arrhythmia
- unstable cardiovascular disease or other unstable medical condition
- uncontrolled arterial hypertension
- recent pulmonary thromboembolism
- upper or lower extremity fracture in the past 3 months

### Randomization and blinding

Included subjects will be randomized by using a randomization list generated electronically (www.randomizer.org) into the combined intervention group and the usual care group (control group) following a simple randomization procedure, in a 1:1 ratio without restriction. Participants will be explicitly informed and reminded not to disclose their randomization assignment to the assessment team, which will be blinded to the participants’ group membership. It will not be possible to conceal the group assignment from the staff involved in the training of the intervention group. Patients (or their families) will be informed of their random inclusion in one group but will not be informed regarding to which group they belong.

### Data collection

**Fig. 1:**
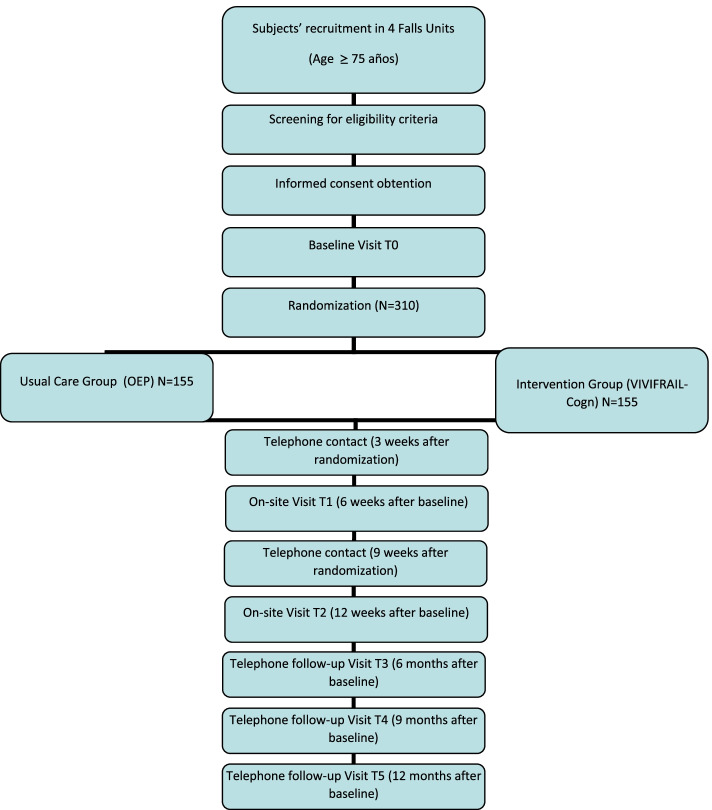
Flow diagram of the study protocol

Data from both the combined intervention and the usual care group will be obtained by the research team (physiotherapist, sport sciences specialist and geriatrician) at three different times: at baseline (T0) and at the 6-week (T1) and 12-week (T2) follow-up visits (Fig. 1). A telephone-based follow-up visit, and a review of the electronic clinical chart will be conducted at 6, 9 and 12 months after the start of the participation for gathering data on falls incidence, functional status and exercise continuation (Table 1). All adverse events, including those related to exercise such as muscle pain, fatigue and general aches and pain, will be recorded in an “adverse events diary” during follow-up visits and telephone calls by the training and testing staff, and by self-report during the study period.

**Table 1:** Study timeline

	**Screening**	**T0** **Basal**	**T1** **6 W**	**T2** **12-W**	**T3** **6 M**	**T4** **9 M**	**T5** **12 M**
***Primary outcome***							
Number of self-reported falls		X	X	X	X	X	X
**Secondary outcomes**							
Risk of falls Usual Gait Speed (UGS) Time up and Go (TUG)	X			X			
Short Physical Performance Battery (SPPB)		X	X	X			
Barthel index (BI)	X		X	X	X	X	X
Frailty Linda Fried criteria	X			X			
Montreal Cognitive Assessment (MoCA)		X	X	X			
Garvan fracture risk calculator		X		X	X	X	X
Yesavage GDS Scale		X		X	X	X	X
UGS with dual tasking		X	X	X			
Clinical criteria of sarcopenia		X		X			
Maximal isometric force of handgrip, knee extension and hip flexion		X	X	X			
Fear of falling (STAC)		X		X	X	X	X
Quality of life (EQ-5D)		X		X	X	X	X
Previous lifestyle		X					
Trail Making Test (TMT – Part A)		X	X	X			
1RM (Leg press)		X	X	X			
Muscular power at 50% 1RM in leg press		X	X	X			
Polypharmacy		X		X	X	X	X
Cumulative Illness Rating Scale for Geriatrics (CIRS-G)		X					
Mini Nutritional Assessment Test (MNA)		X					
Acceleration data: gait kinematic parameters (regularity, variability, cadence), Five Times Sit to Stand Test (peak power, impulse) and balance parameters (power spectrum, area)		X	X	X			
Muscular Echo intensity and thickness (ultrasound)		X		X			
Mortality		X	X	X	X	X	X
Admissions to emergency room and hospitalizations		X	X	X	X	X	X
Institutionalization		X	X	X	X	X	X

The multidisciplinary research team have well-known previous experience in functional geriatric assessment and in the prescription of exercise in frail aged participants in different clinical settings [71, 75, 76].

### Sample size and statistics

Study main outcome is the falls rate/year. For its estimation we will use a negative binomial distribution (over-dispersed Poisson distribution). Assuming an alpha error of α = 5%, an average fall rate of 1,8 falls/year in the usual care group and a mean follow-up of 0.9 years, the sample size required for each group will be 129 to have an 80% power to detect a 30% reduction in the one-year fall rate in the combined intervention group. Considering a withdrawal of 20% during the follow-up, the final simple size per group will be 155. Assumptions are based on results from the Actions Seniors Secondary Falls Prevention in Community Senior Fallers Study [77].

All the participant centers have a remarkable clinical activity attending around 1000 patients every year. It is expected than 15% of these patients in every center can be potentially eligible participants. If not reaching the defined sample size, the inclusion period will be extended for a further 3 months.

Baseline characteristics of the sample will be described by using the central tendency (mean and median) and dispersion (standard deviation, standard error, interquartile ranges and confidence intervals) for continuous variables and absolute (counts) and relative (percentages) frequencies for categorical variables. For comparisons between groups at baseline, t tests or Mann–Whitney U tests will be used for continuous variables, upon normality, which will be checked for each using the Kolmogorov–Smirnov test and normal probability plots, and the chi-square test or Fisher’s test will be used for categorical variables.

Main analyses will focus on the detection of differences in the rate of falling between groups. Study main outcome is the fall rate/year. For its estimation we will use a negative binomial regression (Poisson model extension that allows for the presence of overdispersion). Randomization group will be included as the exposure and factors potentially distorting the estimations as covariates. Robust standard errors consistent with heteroskedasticity will be employed by using the “sandwich” package in R (R 3.4.1 (R Core Team, Vienna, Austria). Falls incident rate ratios and their 95% confidence intervals will be estimated by model coefficients and absolute in the rates will be estimated from the results.

### Data management

Completed personal data or other documents containing protected personal health information will be kept in a locked file at the principal investigator office in every center. Data will be entered into an electronic de-identified database (RedCap) by authorized study team members and checked for completeness and accuracy. Access to data with identifiers will be restricted to authorized study team members and authorities. Electronic data will be kept on a server regulated by the local research institute (IDISNA).

Data will be destroyed 10 years after study finalization or 5 years after last publication.

### Detailed description of the intervention

#### Usual care group (control group)

Participants randomized to the control group will receive normal outpatient care in the Fall Unit. Briefly, it includes a detailed multidimensional assessment performed by the multidisciplinary team (geriatrician, nurse, physiotherapist, occupational therapist) to identify potential intrinsic and extrinsic factors related with falling. Frequently, causality of falling is multifactorial and interventions in the Falls Units are based on the identification of individual risk factors. General approach includes medical review of high risk of falling medications, nutritional interventions like vitamin D in deficient patients, environmental modifications and prescription of the OEP, deemed the gold-standard exercise program in falls management and therefore, part of the usual care delivered to frail older fallers.

Subjects in this group will partake in a home-based exercise program based on the OEP lasting 12-weeks. A physiotherapist will provide a comprehensive explanation of the program, as well as fall risk, safety and adherence information at the baseline visit and will monitor progression during the study visits. Subjects will be encouraged to perform cardiovascular, strength, flexibility and balance exercises 3-times/week. Both volume and intensity will be adjusted by the physiotherapist based on participant’s self-reported feelings. Subjects will be provided with a booklet with instructions (Additional File 1) and an adherence log (Additional File 2) that will be filled in by the participant or a proxy.

#### Intervention group (VIVIFRAIL-COGN)

Participants assigned to this group will participate in a 12-week home-based program combining the VIVIFRAIL multicomponent physical exercise and an individualized cognitive training programme (VIVIFRAIL-COGN).

- B1- Multicomponent VIVIFRAIL exercise group

The multicomponent physical exercise program will be based on the VIVIFRAIL programme which was developed in Europe (Erasmus + programme of the European Union).

The VIVIFRAIL multicomponent exercise intervention program includes individualized sets of resistance training, gait and balance training, which appear to be the best strategy for falls rate reduction in older individuals and to maintaining functional capacity during ageing [78]. This type of intervention has also been proven as the most effective to delay disability, cognitive impairment and depression [79] as well as effective to reverse the functional decline associated with acute hospitalization in very old patients [71]. The VIVIFRAIL exercise program was originally designed with the aim of providing guidance in exercise prescription to professionals managing frail older adults with or without falls older than 70 years [78]. The programme is tailored to the older person’s functional capacity evaluated by the SPPB and a UGS test and the risk of falling, aligning with the new functional-based older adult’s health perception proposed by the WHO [70] Different standardized programs oriented to the functional level of the individual were designed and description of the performance, intensity, volume, frequency and monitoring are described in the materials published. Recently, a new user-friendly simplified version and a simple mobile app with interactive videos have been produced to ease adoption and maximize adherence. (see www.vivifrail.com).

In VIVIFRAIL, subjects are assessed before the start of participation to rule out the presence of any contraindication to exercise (see above). Afterwards, functional capacity is assessed by means of physical performance tests to classify the older individual into one of the four exercise programs proposed. These physical performance tests are the SPPB and the 6-m UGS test (Table 2) with each leading to the recommendation of a certain customized multicomponent physical exercise program (see Table 2). If a high risk of falling is detected by the presence of recurrent falls (≥ 2 falls in the previous year or at least one fall requiring medical attention), reduced performance in the Up-and-Go Test (≥ 20 s) or the UGS (< 0.8 m/s) or a diagnosis of dementia, the balance component of the program is reinforced (indicated by the addition of “ + ” to the program name).

**Table 2:** Exercise programs based on individual functional status assessed through the physical performance-based tests

SPPB Score	6-m UGS Test	Exercise Program^a^
0–3	< 0.5 m/s	A-Disability
4–6	0.5–0.8 m/s	B-Frailty
7–9	0.9–1 m/s	C-Pre-frailty
10–12	> 1 m/s	D- Robustness

^a^If a high risk of falling is detected: TUG (≥ 20 s), UGS (< 0.8 m/s), diagnosis of dementia and/or recurrent falls/falls requiring medical attention in the last year, subjects will be assigned to the “ + ” program, making emphasis on balance training

Once the individual has been assigned to an exercise program, they will partake in a 12-week exercise program with a frequency of 3 sessions/week. All the programs in Vivifrail share the following characteristics:

- Resistance training:

▪ Sets and repetitions

Subjects will be encouraged to perform 3-4 exercises (depending on the program), with a volume of 3 series of 12 repetition. A 1 to 3 minute-break will be encouraged between exercises.

▪ Intensity and progression:

The intensity will be that allowing the completion of all the series with a feeling of moderate effort at the end of each series. Resistance will be adjusted accordingly along the duration of the program.

- Cardiovascular training:

▪ Volume

Walking activities-based cardiovascular training will be recommended to improve cardiorespiratory fitness. Depending on the functional capacity of the individuals, walking bouts ranging from 5-10 seconds to 45 minutes will be prescribed.

▪ Intensity and progression

Subjects will be instructed to maintain a moderate effort during walking bouts and to progressively increase both the duration and the cost of walking.

- Balance training:

Balance training will include both static and dynamic exercises adapted to individual abilities of the person.

▪ Volume

During static exercises, subjects will be instructed to remain in the same position for ten seconds in different conditions (one-foot stand, semi-tandem stand with eyes open/closed or in unstable surfaces). After resting, they will repeat three times.

In the case of dynamic exercises, subjects will perform different tasks (walking over obstacles, in different directions, paying attention to external stimulus…) in different conditions that should challenge their balance by demanding postural corrections. They will repeat the task 2-3 times.

In the case of the presence of high risk of falling (see above) the number, frequency and volume of balance exercises is increased.

▪ Intensity and progression

Subjects will progress towards more challenging situations (both in terms of feature of the exercises, difficulty or conditions) when current exercises do not require enough effort from them.

Safety recommendations will be provided to prevent falls during balance exercises.

- Flexibility

Subjects will be instructed to perform a series of stretching exercises at the end of the sessions.

▪ Volume

They will perform 2-3 stretching exercises, with 2-3 repetitions per exercise lasting 10 seconds. They will be told to stretch until they feel a bit of tension and remain in the same position without discomfort.

Materials will be provided to allow correct execution and progression of the exercises and to maximize adherence. The latest Vivifrail update includes a passport-like booklet that allows to follow the session and have access to the graphical and textual descriptions of the exercises, as well as information relative to volume, frequency, intensity, and duration. In addition, it includes a section for registering adherence and the adherence log, respectively (see www.vivifrail.com).

During the baseline visit, the participant and a proxy will be informed about the features of the program and potential side effects, as well as safety and adherence measures and booklet functioning. The research team will also provide adaptable ankle weights.

- B2-Cognitive training

The cognitive training program will last 12 weeks as well and will consist in twice weekly sessions combined with the above-mentioned physical exercise program (Table 3). Before the start of the intervention, cognitive status will be assessed through a global cognition test -Montreal Cognitive Test (MoCA) [80]- in order to individualize cognitive training exercises with the aim of maximizing benefits. The MoCA is a screening test with scores from 0 (worse cognitive function) to 30 (better cognitive function) and assesses different cognitive functions (including executive functions). In addition, adds other type of tasks as the clock test, a part of the Trail Making Test (part B), verbal fluency and denomination, items normally evaluated in a general assessment of cognition. For all these reasons the MoCA test is consider a more complete and sensitive screening test in older adults to monitor intervention than Mini-mental State Examination( MMSE) [81].

**Table 3:** Within cognitive session structure:

Session = Set A + Set B
**Set A:** Set of attentional activities: A.1.) An exercise of sustained or selective attention with visual or auditive stimulus An exercise of dual or divided attention A.2.) An exercise of sustained or selective attention with visual or auditive stimulus An exercise of alternating attention
**Set B:** Set of executive function activities: B.1.) Processing speed and preceptive organization B.2.) Working memory and high attention system B.3.) Comprehension, abstraction and verbal reasoning B.4.) Planification, execution and problem solving (decision making and temporary estimation)

Based on the score of the MoCA and normative values in the Spanish population over 75 [81] years old, subjects will receive an intervention from the following:

- Program A. MoCA 26–30. High cognitive performance. Normal cognitive function
- Program B. MoCA 20–25. Intermediate cognitive performance. No evidence of clinical cognitive decline
- Program C. MoCA 13–19. Low cognitive performance. Mild cognitive decline
- Program D. MoCA ≤ 12. Very low cognitive performance. Mild dementia

In every session a combination of one exercise from Set A (attentional activities) and one exercise from Set B (executive function activities) will be performed (see Table 3). An initial training session will be performed to explain the programme to the participants and their relatives or caregiver (the support of one person during sessions will be needed due to characteristics of the cognitive training exercises).

To guarantee adherence to the program and appropriate execution of cognitive training exercises, a videoconference session will be performed at week 3 and 9 of the program by the occupational therapists. The purpose of this monitoring is to resolve doubts, adapt difficulty and provide support.

Either the participant and the companion will be instructed in the general and individual characteristics of both physical and cognitive programs (VIVIFRAIL-COGN). Also, all participants will receive instructions, printed materials and audiovisual support of the cognitive sessions with the VIVIFRAIL-COGN material.

Table 4 shows the recommended distribution of both the physical and cognitive components of the intervention. Adherence to the cognitive training program will be registered together with the exercise in the VIVIFRAIL booklet.

**Table 4:** Recommended distribution of exercise and cognitive training sessions per week

	**Monday**	**Tuesday**	**Wednesday**	**Thursday**	**Friday**	**Saturday**	**Sunday**
Morning	VIVIFRAIL Wheel	Walk	VIVIFRAIL Wheel	Walk	VIVIFRAL wheel	Walk	Rest
Afternooon		Cognitive training		Cognitive training			

### Outcome measures

#### Primary outcome

The primary outcome is the change in the number of auto-reported falls during the study period. Falls, number of falls and its consequences will be registered by the participants /relatives /caregivers with a diary register according with ProFANE group (Prevention of Falls Network Europe) recommendations on reporting of fall prevention programs’ outcomes [62, 82]. Participants/relatives/caregivers will be instructed to use the diary register (Additional File 3) and will be reviewed by the investigator team during T0, T1 and T2 assessments. During follow-up (6, 9 and 12 months after the start of the intervention) a telephone call and a review of the electronic clinical chart will be conducted to register falls and associated events.

#### Secondary outcomes

The secondary measures will be assessments that evaluate fall-related constructs such as fall-related healthcare system utilization, cognitive and physical capacities, psychological and muscle size and quality by echography. Additionally, instrumented measurements of frailty-related kinematic parameters will be performed during the physical performance measures by using inertial sensors (Table 1).

#### Fall-related outcomes

In addition to fall occurrence along 1-year follow-up, in this study, we will assess a set of core outcomes recommended by the ProFANE initiative and that have been poorly reported in previous studies [68, 82]. During follow-up and telephone visits, fall-related healthcare system use (contacts, admissions, emergency visits) and central/peripheral fall-related fracture (radiologically confirmed) will be ascertained and later confirmed by medical records check. In addition, fall related psychological consequences will be assessed through the assessment of the presence of fear of falling [83]. Health-related quality of life (HRQoL) will be measured through the Spanish version of the EuroQoL-5D [84]. Physical activity levels will be evaluated through the Spanish version of the Brief Physical Activity questionnaire [85].

### Fall risk

An increased risk of falling will be considered according to national and international recommendations (one or more of the following criteria): 2 or more falls or 1 fall with medical attention in the last twelve months or a significative gait disorder assessed by performance tests (TUG > 20 s and or UGS test < 0.8 m/s) [14, 86]. The TUG test is a classic test to assess risk of falls. Participant must raise from a chair without help, walk three meters, turn back and sit again. A time of > 20 s is indicative high risk of falls [87].

### Physical function endpoints

Physical function of the participants will be evaluated by the SPPB [88], which evaluates, balance, gait ability and leg strength using a single tool. Total score ranges from 0 (worst) to 12 points (best). The SPPB validity for frailty screening and predicting disability, institutionalization and mortality. A total score of less than 10 is deemed indicative of frailty and a high risk of disability and falls. A 1-point change in SPPB is deemed a meaningful clinical change [89].

Isometric handgrip strength will be measured using a manual dynamometer in two attepms in both dominant and non-dominants hands. The best of both will be recorded. Handgrip asimmetry has recently been proposed as a early marker of future falls [90].

Maximal dynamic lower limb strength (in kilograms) will beassessed using the 1 repetition maximum (1RM) test in the bilateral leg press exercise using exercise machines (Matrix, Johnson Health Tech, Ibérica, S.L., Torrejón de Ardoz, Spain; and Exercycle S.L., BH Group, Vitoria, Spain). In the first assessment, subjects will be instructed on the exercise execution and they will warm-up with specific movements for the leg press. Subject’s maximal load will be established in no more than five attempts, with a recovery period lasting 3 min between attempts. After the 1RM values are determined, the maximal power output will be measured (in watts) by the performance of 10 repetitions at maximal velocity at an intensity of 50% of 1RM by using a linear velocity transducer connected to the weight plates (Vitruve linear Encoder System,Vitoria, Spain). During all neuromuscular performance tests, strong verbal encouragement will be given to each subject to motivate them to perform each test actionas optimally and rapidly as possible.

### Sarcopenia-related measures

Sarcopenia clinical probability will be measured using the definition proposed by the European Working Group on Sarcopenia in Older People (EWGSOP) [91]. In these criteria clinical suspiction is present in subjects with a > 4 in the SARC-F scale, and probable sarcopenia is confirmed with a 5-repetition chair test time > 15 s or a dominant handgrip strength < 27 kg in men or < 16 kg in women. Muscle quality (echo-intensity) and size (muscle thickness) of the rectus anterior muscle and eminence thenar will be assessed with a portable ultrasound system (Philips Healthcare Lumify C5-2 Transductor, Philips Ultrasound, Bothell, WA,USA) operated by a trained member of the researcher’s team [92].

### Fracture risk

Risk of fracture will be assessed with the Garvan fracture risk calculator (https://www.garvan.org.au). This tool allows to estimate 5- and 10-years fracture risk by equations based on anthropometric data, previous fractures since the age of 50, falls in the last year and optionally bone mineral density data. It has been validated very recently in Spanish population [93].

### Neuropsychological assessment

The MoCA [80] test will be used to assess changes in cognitive function and to individualize cognitive intervention. MoCA has been explained in detail before (see paragraph B2). The Trail Making Test (TMT), part A, will be used to assess executive function. In TMT-A, the participant is asked to connect randomly arranged circles containing numbers from 1 to 25 following the number sequence, and doing it as quickly as possible [94]. Mood will be assessed through the Yesavage GDS [95], a 15-item scale used to screen for depressive symptoms in older adults.

### Dual-task GS

Worsening of the performance in the GS while performing a dual-task test (dual-task cost) may be early predictors of fall risk, and may be useful tools for functional evaluations in frail older patients. The dual-task paradigm will be used in the 5-m usual GS. Two trials will be conducted to assess GS while the patient is performing a verbal fluency and a counting task (verbal GS and arithmetic GS, respectively) [96, 97]. Participants will be instructed to walk one trial over 5-m as a single-task.

During the verbal dual-task condition (verbal GS), we will measure GS while participants are naming animals aloud. During the arithmetic dual task condition (arithmetic GS), we will assess GS while participants are counting backwards aloud from 100 in ones. Besides the dual task-cost (relative increase in time to complete the 5-m walk under dual-task conditions with respect to single-task), the cognitive accuracy will be measured by counting the number of animals named (dual-task with verbal performance) or determining how many numbers were correctly counted backwards (dual-task with arithmetic performance).

### Kinematic parameters assessment

During functional tasks (such as balance, gait and rising from a chair) and dual task walk, an inertial sensor unit (XSENS, Xsens Technologies B.V. Enschede, Netherlands) will be attached over the lumbarspine (L3) to record raw acceleration data. Afterwards, the raw signal we be processed to compute kinematic parameters related to physical frailty [98–100] by using the software designed by Movalys (Movalsys SL, Pamplona, Spain).

### Study protocol

The protocol employs relevant standard protocol items for clinical trials according to the SPIRIT 2013 statement (Additional File 4) [29] and follows the CONSORT statement [30] for transparent reporting. The trial is registered at ClinicalTrials.gov (ID number NCT04911179), and the status is on recruitment.

## Discussion

The main objective of the proposed study is to assess the efficacy of VIVIFRAIL-COGN, a program that combines the individualized multicomponent exercise plan-VIVIFRAIL- with an individualized cognitive training focused on improving executive function to reduce auto-reported falls rates and risk of falling, compared with a standard intervention. To our knowledge, this is the first trial that will investigate the direct effect of combined physical exercise with cognitive training on falls in a frail population of older persons with high risk of falling.

We have just started the decade of Healthy Ageing 2020–2030, an important opportunity to bring together governments, civil society, international agencies, professionals, academia, the media, and the private sector for ten years of concerted, catalytic and collaborative action to improve the lives of older people, their families, and the communities in which they live [101]. Healthy ageing is defined by the World Health Organization (WHO) as the maintaining of functional capacity, recently renamed as intrinsic capacity, as the central objective and focus of all health systems and policies for elderly populations [70]. Among 10 priority strategies to develop in this decade, WHO highlights (priority 4) to promote research that addresses the current and future needs of older people [102]. Our study is perfectly aligned with this strategy. Actually, falls constitute an extraordinary prevalent geriatric syndrome. Globally, a third of people aged 65 years and older fall at least once per year, with 5% of these falls resulting in a fracture [103]. WHO considers falling as a major public health problem and points out the importance to prioritize specific research [104]. Thus, the clinical impact of our study can be significant given its potential to help change the actual healthcare paradigm of frail patients to a more centered and functional perspective.

Nowadays healthcare actions to prevent falls in frail older persons with high risk of falling remain scarce, isolated and usually fragmented, despite the efforts that are being made by policymakers, reflected in the recent availability of evidence-based public national/international recommendations [12, 73, 86]. In those recommendations, physical exercise appears as the most evaluated intervention. It is very well known that it is an effective and safe intervention to reduce risk and rate of falls [37, 41, 42]. Our research group very recently showed that the VIVIFRAIL program (www.vivifrail.com) [75] is feasible and effective to improve functional capacity in frail older persons with cognitive problems [72] but we did not explore its potential in falls prevention. It is possible that more integrated and multidomain interventions, as proposed in the present study protocol, are needed in the case of frail older persons. Specifically, if our hypothesis is correct and we show that VIVIFRAIL-COGN intervention is more effective in terms of rate, risk of falls and functional improvement in this population, a new therapeutic window is opened: falls and risk of falls may be improved (and potentially the burdensome consequences of falls, such as fractures) through combined physical-cognitive interventions.

One of the strengths of our study is the multidisciplinary nature of the project (geriatricians, physiotherapists, occupational therapists, psychologists, nurses). This is a remarkable opportunity to establish new protocols and share our results with the scientific community and healthcare professionals with interest in frail older persons with high risk of falling.

Another important aspect of our study is the non-exclusion of frail older patients with cognitive problems. Falls are very prevalent in cognitive decline frail older persons and cognitive decline it is a very well-known risk factor of falling and fractures. The non-exclusion of frail participants with high risk of falling and cognitive problems improves external validity and makes the trial unique in comparison with previous trials in this research area.

In summary, this trial will provide new evidence about the effectivity of an individualized multidomain interventions on falls by studying the effect of additive effects of cognitive training and physical exercise to prevent falls in older frail persons with high risk of falling.

### Dissemination

We will disseminate the results of our study via presentations at international conferences and articles in peer reviewed journals. The study will be carried out and reported in following the Standard Protocol Items: Recommendations for Interventional Trials (SPIRIT) guidelines.

### Future directions

This project offers the opportunity to test a novel multidomain intervention (VIVIFRAIL-COGN). Following the VIVIFRAIL methodology of individualize physical exercise prescription according to functional status , this intervention is a step forward to prescribe more individualized and person-centered interventions of frail older persons with high risk of falling. We expect to translate this methodology to others Falls Units in Spain and in the next future disseminate this integrative and novel model to other clinical geriatric and non-geriatric units attending these patients. Finally, we expect to translate our model to rutinary clinical practice and as a global strategy to implement in the Spanish Governmental Strategy of Falls and Frailty.

### Trial status

The trial began recruitment in July 2021 and is currently open for recruitment. Recruitment will cease when 310 participants have been randomized. It is anticipated that this target will be reached by September 2023.

## Supplementary Information

**Additional file 1. **Protocol contributors.

**Additional file 2. **OTAGO adherence log.

**Additional file 3. **Falls log.

**Additional file 4. **Spirit checklist. 

## Data Availability

Individual deidentified participant data (including data dictionaries) that underline the results reported in this article (text, tables, figures and appendices) will be shared. Other documents as Study Protocol, Statistical Analysis Plan and Analytic code will be available. Data will be become available beginning 3 months and ending 5 years following article publication. Access criteria data will be shared to researchers who provide a methodologically sound proposal to achieve aims in the in the approval proposal. Proposals should be directed to alvaro.casas.herrero@navarra.es. Data are available for 5 years at a thirdly party website ( www.idisina.es).

## Abbreviations

BI: Barthel Index
EQ-5D: EuroQoL-5D
EWGSOP: European Working Group on Sarcopenia in Older People
GDS: Geriatric Depression Scale
GS: Gait Speed
HR-QoL: Health-related Quality of Life
MNA: Mini-Nutritional Assessment
MoCA: Montreal Cognitive Assessment
MMSE: Mini-Mental State Examination
OEP: Otago Exercise Program
ProFANE: Prevention of Falls in Europe
RM: Repetition Maximum
SPPB: Short Physical Performance Battery
STAC: Síndrome de Temor a Caerse
TMT: Trail Making Test
TUG: Up-and-Go Test
UGS: Usual Gait Speed
WHO: World Health Organization
