# Correction: Effect of a multicomponent exercise program and cognitive stimulation (VIVIFRAIL-COGN) on falls in frail community older persons with high risk of falls: study protocol for a randomized multicenter control trial

**DOI:** 10.1186/s12877-022-03535-0

**Published:** 2023-01-19

**Authors:** Juan Luis Sánchez-Sánchez, Cristina Udina, Almudena Medina-Rincón, Mariano Esbrí-Victor, Irene Bartolomé-Martín, Débora Moral-Cuesta, Itxaso Marín-Epelde, Fernanda Ramon-Espinoza, Marina Sánchez-Latorre, Fernando Idoate, Adriana Goñi-Sarriés, Blanca Martínez-Martínez, Raquel Escudero Bonet, Julián Librero, Álvaro Casas-Herrero

**Affiliations:** 1 MOVE-IT Research Group, Department of Physical Education, Faculty of Education Sciences, University of Cadiz, Cadiz, Spain; 2 Health Sciences Department, Universidad Pública de Navarra (UPNA), Pamplona, Spain; 3 Institut de Viellissement, CHU Toulouse, Gerontopole de Toulouse, Toulouse, France; 4 Parc Sanitari Pere Virgili, Barcelona, Spain; 5 RE-FiT Bcn Research Group, Vall Hebron Research Institute, Barcelona, Spain; 6 Geriatrics Department, Complejo Hospitalario Universitario de Albacete (CHUA), Albacete, Spain; 7 Geriatrics Department, Hospital Universitario de Guadalajara (HUG), Guadalajara, Spain; 8 Geriatrics Department, Hospital Universitario de Navarra (HUN), C/Irunlarrea s/n 31008, Pamplona, Spain; 9 Functional Recovery Unit, Hospital San Juan de Dios (HSJD), Pamplona, Spain; 10 Radiology Department, Mutua Navarra, Pamplona, Spain; 11 Mental Health Network, Navarra Healthcare System, Pamplona, Spain; 12 Navarra Institute for Health Research (IdiSNA), Pamplona, Spain; 13 CIBER of Frailty and Healthy Aging (CIBERFES), Instituto de Salud Carlos III, Madrid, Spain

**Correction: BMC Geriatrics 22, 612 (2022)**

**https://doi.org/10.1186/s12877-022-03214-0**

After publication of this article [1], the authors reported that the statement in the Funding information section was incorrectly given as 'The present study is funded by a grant from the Spanish Ministry of Science and Innovation and the Instituto de Salud Carlos III (PI20/01546).' and should have read

'The present study is funded by a grant from the Spanish Ministry of Science and Innovation and the Instituto de Salud Carlos III (PI20/01546), co-financed by FEDER funding'.

The original article [1] has been corrected.
