# Supplementary material for: Effect of a multicomponent exercise program and cognitive stimulation (VIVIFRAIL-COGN) on falls in frail community older persons with high risk of falls: study protocol for a randomized multicenter control trial
Source: BMC Geriatr. 2022 Jul 23;22:612. doi: 10.1186/s12877-022-03214-0 (PMC9308197; doi:10.1186/s12877-022-03214-0)
Supplement: Supplementary file 2 — Additional file 2. OTAGO adherence log. [file 12877_2022_3214_MOESM2_ESM.docx]

**EXERCISE ADHERENCE LOG-OTAGO PROGRAM Participant’s ID: ____________________**

Week 7-12

Please, register days of participation in the exercise program. Indicate any adverse event/difficulties when performing exercise.

| Monday __/___/_____ | Tuesday __/___/_____ | Wednesday __/___/___ | Thursday __/___/_____ | Friday __/___/_____ | Saturday | Sunday |
| --- | --- | --- | --- | --- | --- | --- |
| Monday __/___/_____ | Tuesday __/___/_____ | Wednesday __/___/___ | Thursday __/___/_____ | Friday __/___/_____ | Saturday | Sunday |
| Monday __/___/_____ | Tuesday __/___/_____ | Wednesday __/___/___ | Thursday __/___/_____ | Friday __/___/_____ | Saturday | Sunday |
| Monday __/___/_____ | Tuesday __/___/_____ | Wednesday __/___/___ | Thursday __/___/_____ | Friday __/___/_____ | Saturday | Sunday |
| Monday __/___/_____ | Tuesday __/___/_____ | Wednesday __/___/___ | Thursday __/___/_____ | Friday __/___/_____ | Saturday | Sunday |
| Monday __/___/_____ | Tuesday __/___/_____ | Wednesday __/___/___ | Thursday __/___/_____ | Friday __/___/_____ | Saturday | Sunday |
