# Supplementary material for: Effect of a multicomponent exercise program and cognitive stimulation (VIVIFRAIL-COGN) on falls in frail community older persons with high risk of falls: study protocol for a randomized multicenter control trial
Source: BMC Geriatr. 2022 Jul 23;22:612. doi: 10.1186/s12877-022-03214-0 (PMC9308197; doi:10.1186/s12877-022-03214-0)
Supplement: Supplementary file 3 — Additional file 3. Falls log. [file 12877_2022_3214_MOESM3_ESM.docx]

Participant ID:

ASSESSMENT OF THE EFFECTIVENESS OF A COMBINED INDIVIDUALIZED PHYSICAL EXERCISE AND COGNITIVE TRAINING TO MANAGE FALLS IN FRAIL OLDER ADULTS WITH HIGH FALLING RISK

FALLS LOG

Date follow-up begin: ____/____/___________


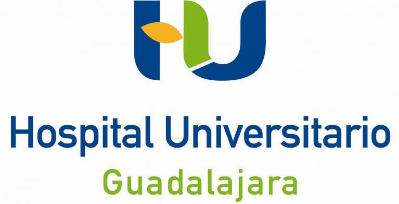

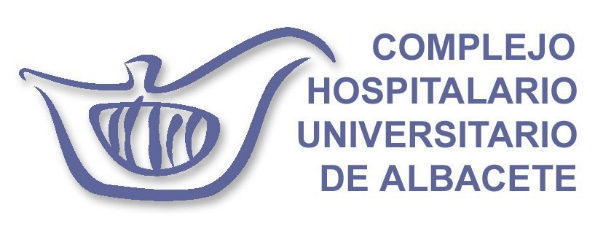

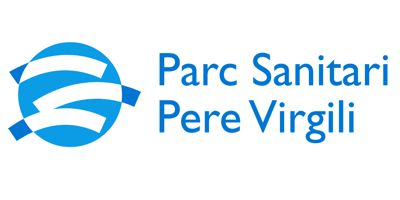

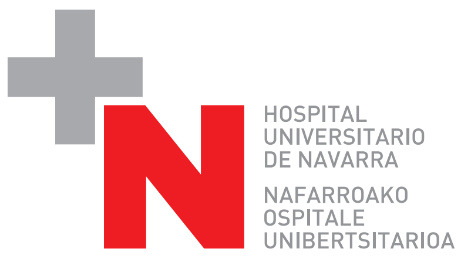


**FALLS LOG**

In the context of the study you are participating in, it is of high relevance to register the number and consequences of falls that you might experience during the follow-up. By having this valuable information, we can better understand the how and why falls occur and try to reduce their number and impact. qué ocurren las caídas y de esa manera podremos intentar ponerle solución.

In the case you suffer an event susceptible of being considered a fall, we kindly ask you to register it in the present log, indicating the date, whether it required medical attention and the consequences of this event such as lacerations or fractures.

In the odd case you suffer a fall, please, sought for help or try to handle a stable piece of furniture. Check if you are injured and call the emergency phone number (112) if it is the case or feel unsafe.

**Instructions to fill the log in:**

Fill a gap for every fall you might experience. In the case face difficulties when doing it, ask a relative or proxy to assist you.

**REGISTRO CAÍDAS**

**Fall number 1**

**Date**

**Context**

**Which do you think was the reason of your fall?**

**Did you require medical attention?**

**Fall number 2**

**Date**

**Context**

**Which do you think was the reason of your fall?**

**Did you require medical attention?**

**Fall number 3**

**Date**

**Context**

**Which do you think was the reason of your fall?**

**Did you require medical attention?**

**Fall number 4**

**Date**

**Context**

**Which do you think was the reason of your fall?**

**Did you require medical attention?**

**Fall number 5**

**Date**

**Context**

**Which do you think was the reason of your fall?**

**Did you require medical attention?**

**Fall number 7**

**Date**

**Context**

**Which do you think was the reason of your fall?**

**Did you require medical attention?**

**Fall number 6**

**Date**

**Context**

**Which do you think was the reason of your fall?**

**Did you require medical attention?**

**Fall number 8**

**Date**

**Context**

**Which do you think was the reason of your fall?**

**Did you require medical attention?**

**Fall number 9**

**Date**

**Context**

**Which do you think was the reason of your fall?**

**Did you require medical attention?**

**Fall number 10**

**Date**

**Context**

**Which do you think was the reason of your fall?**

**Did you require medical attention?**

**Fall number 11**

**Date**

**Context**

**Which do you think was the reason of your fall?**

**Did you require medical attention?**

**Fall number 12**

**Date**

**Context**

**Which do you think was the reason of your fall?**

**Did you require medical attention?**
